# Preclinical Screening for Retinopathy of Prematurity Risk Using IGF1 Levels at 3 Weeks Post-Partum

**DOI:** 10.1371/journal.pone.0088781

**Published:** 2014-02-11

**Authors:** Alejandro Pérez-Muñuzuri, Ma Luz Couce-Pico, Ana Baña-Souto, Olalla López-Suárez, Alicia Iglesias-Deus, José Blanco-Teijeiro, José Ramón Fernández-Lorenzo, José María Fraga-Bermúdez

**Affiliations:** 1 Neonatology Service, Department of Paediatrics, Clinical Hospital of the University of Santiago de Compostela, Santiago de Compostela, Spain; 2 Ophthalmology Service, Clinical Hospital of the University of Santiago de Compostela, Santiago de Compostela, Spain; University of Sydney, Australia

## Abstract

Following current recommendations for preventing retinopathy of prematurity (ROP) involves screening a large number of patients. We performed a prospective study to establish a useful screening system for ROP prediction and we have determined that measuring serum levels of IGF1 at week three and the presence of sepsis have a high predictive value for the subsequent development of ROP. A total of 145 premature newborn, with birthweight <1500 g and/or <32 weeks gestational age, were enrolled. 26.9% of them showed some form of retinopathy. A significant association was found between the development of retinopathy and each of the following variables: early gestational age, low birthweight, requiring mechanical ventilation, oxygen treatment, intracranial haemorrhage, sepsis during the first three weeks, bronchopulmonary dysplasia, the need for erythrocyte transfusion, erythropoietin treatment, and low levels of serum IGF1 in the third week. A multiple logistic regression analysis was used to obtain curves for the probability of developing ROP, based on the main factors linked with ROP, namely serum levels of IGF1 and presence of sepsis. Such preclinical screening has the ability to identify patients with high-risk of developing retinopathy and should lead to better prediction for ROP, while at the same time optimising the use of clinical resources, both human and material.

## Introduction

Retinopathy of prematurity (ROP) is a retinal vascular disease affecting premature babies which, without early identification and treatment, can lead to retinal detachment and loss of vision. ROP is one of the main causes of blindness in premature babies [1],[2].

Multiple pathogenic factors have been identified for ROP. Important among these are an association with blood oxygen saturation and with infection [3],[4],[5],[6]. Recently, insulin-like growth factor 1 (IGF1) has been shown to play a role in the pathophysiology of this retinopathy. Specifically, a deficit of IGF1 in the first few weeks of post-natal life pre-dispose to non-proliferative stages of the disease, while a subsequent increase in IGF1 levels can lead to proliferative stages of ROP [7],[8],[9].

The necessity for early diagnosis and the establishment of a set of clinical indications for laser photocoagulation treatment, which reduces the incidence of retinal detachment and substantially reduces morbidity in ROP, have led to recommendations by the American Academy of Pediatrics with respect to ocular examination in premature babies [10],[11]. Thus, babies with a birthweight of 1500 g or less, or gestational age of 32 weeks or less, or birthweight between 1500 g and 2000 g, or more than 32 weeks, who either have an unstable clinical progression or who require respiratory support, should all undergo ocular examination. Indications for treatment have been established in the CRYO-ROP study [12] and for laser in the ETROP study [13].

Current guidelines include a large group of patients and the beginning of examinations varies according to gestational age but on average is between 4 and 6 weeks after birth. As this requires not only the allocation of a dedicated team and significant resources, but also the early identification of at-risk patients, several screening methods have been developed, all of which are based primarily on post-natal weight gain [14],[15],[16].

We have recently shown an important relationship between the development of ROP and serum levels of IGF1 measured in the 3rd week after birth which thus constitute a biomarker for the disease. Additionally, the association was strengthened in the presence of neonatal infection in the first three weeks defined as clinical, analytical and/or microbiological criteria [9].

The aim of the current study was to establish a method of preclinical screening for ROP, in patients of less than 32 weeks gestational age and/or birthweight of less than 1500 g, based on the following criteria: serum IGF1 levels and/or presence of clinical, analytical or microbiological sepsis in the 3rd week post-partum, gestational age and birthweight.

## Materials and Methods

We carried out a prospective study over a 5-year period (2006–2011) on newborn premature babies of birthweight less than 1500 g and/or less than 32 weeks gestational age (as determined by the Dubowitz/Ballard morphological maturation scoring system [17],[18],[19] in conjunction with the date of birth estimated from the 12-week ultrasound).

157 in-patients meeting the above criteria were evaluated –first 74 were previously reported [9]-. The hospital is a tertiary referral care centre for a population of 394,172 and the Neonatal Unit has Level 3b status. After applying the following exclusion criteria a total of 145 patients were recruited for the study: gestational age less than 24 weeks, any congenital abnormality, death, or absence of informed consent from the parents. The study was approved by the Clinical Hospital of the University of Santiago de Compostela Ethics Committee and a written informed consent was obtained from the next of kin. In each patient included in the study, serum level of insulin-like growth factor (IGF1) was measured in the third week after birth -it was described that IGF1 levels at third week have more sensitivity and specificity on detection ROP disease [9]-. 1.2 ml of blood was obtained by peripheral venipuncture, serum obtained by centrifugation and analysis carried out using an enzyme-labelled chemiluminescent immunometric assay (IMMULITE 2000 IGF-I, Siemens (R) Healthcare Diagnostics Ltd., Surrey, UK).

Other factors associated with ROP were also evaluated: gestational age, morphometric data at birth, type of birth, mechanical ventilation (type and duration), oxygen (fraction of inspired oxygen (FiO_2_) and duration), sepsis in the first three weeks, bronchopulmonary dysplasia, patent ductus arteriosus, intracranial haemorrhage, need for erythrocyte transfusion or treatment with erythropoietin (EPO).

The ocular examination was carried out by an ophthalmologist trained specifically in the diagnosis of retinopathy of prematurity, and performed between the 4th and 6th weeks post-partum or, if it occurs previously, at 34 weeks of corrected gestational age. Ocular examinations were continued until the patient was discharged by the ophthalmologist.

Statistical analyses were carried out using the SPSS® 15.0 Windows® program. Student's t-test, U Mann-Whitney test and the chi-squared test were used for quantitative (normalized or not) and qualitative variables, respectively. A multiple logistic regression analysis was carried out on variables found to be associated with ROP, and curves obtained to show the probability of developing the disease. A two tailed ‘p’ value of less than 0.05 was considered statistically significant.

## Results

Of the 145 patients recruited to the study, 39 (26.9%) developed some degree of ROP. Of these, 20 developed ROP stage 1 (51.3%), 12 stage 2 (30.8%) and 7 stage 3 (17.9%). 7 patients had ROP-plus disease (17.9%). Photocoagulation treatment was indicated only in 5 (12.8%) patients.

Measured variables are shown in Table 1. Patients who developed ROP were of significatively lower gestational age and birthweight, and shorter body length and cranial circumference at birth. No significant differences were seen with respect to gender, type of birth, antenatal steroid treatment, multiple birth or necessity for treatment with surfactant.

**Table 1:** Demographics and characteristics of enrolled patients (mean ± standard deviation).

			*ROP*		
			*No*	*Yes*	p
***Number of patients***			106	39	
***Gestational age at birth (weeks)***			30.96±1.98	28.99±2.02	0.000
***Birthweight (g)***			1361.17±263.19	1094.05±224.71	0.000
***Length at birth (cm)***			38.87±2.67	36.77±2.59	0.000
***Cranial circumference at birth (cm)***			27.56±3.01	26.01±1.91	0.000
***Sex***	Female		56 (52.8%)	14 (35.9%)	NS
	Male		50 (41.2%)	25 (64.1%)	
***Type of birth***	Vaginal		24 (22.6%)	7 (17.9%)	NS
	Cesarean		82 (77.4%)	32 (82.1%)	
***Antenatal steroids***			77 (72.6%)	25 (64.1%)	NS
***Multiple birth***			37 (34.9%)	10 (25.6%)	NS
***Surfactant treatment***			52 (49%)	23 (58.9%)	NS
***Mechanical ventilation***			74 (69.8%)	35 (89.7%)	0.014
	HFO		3 (2.8%)	6 (15.4%)	0.005
	Conventional		43 (40.6%)	22 (56.4%)	NS
		Number of days	1.41±3.76	3.81±6.51	0.035
	Non-invasive		70 (66.1%)	35 (89.7%)	0.005
		Number of days	3.76±5.13	11.31±11.35	0.000
***Oxygen administration***			59 (55.7%)	32 (82.1%)	0.004
	Number of days		5.15±9.76	17.19±22.63	0.003
	Maximum FiO_2_		0.31±0.14	0.39±0.21	0.028
***Intracranial haemorrhage***			16 (15.1%)	16 (41%)	0.001
***Sepsis (<3 weeks post-partum)***			32 (30.2%)	30 (76.9%)	0.000
***Patent ductus arteriosus***			19 (17.9%)	12 (30.8%)	NS
***Bronchopulmonary dysplasia***			10 (9.4%)	16 (41%)	0.000
***Blood transfusion***			13 (12.3%)	19 (48.7%)	0.000
***EPO treatment***			47 (44.3%)	27 (69.2%)	0.008
***IGF1 (3^rd^ week)***			44.5±14.93	28.97±7.23	0.000

Abbreviations: FiO_2_: fraction of inspired oxygen; HFO: high-frequency oscillatory ventilation.

A total of 109 patients required some form of respiratory support. A significantly higher incidence of ROP was found in patients receiving high-frequency oscillatory ventilation, and in those ventilated for longer, either conventionally or non-invasively.

Oxygen administration, including longer-term and with a higher FiO_2_, were also significantly correlated with the development of ROP.

A clear association was found between ROP and intracranial haemorrhage, sepsis in the first three weeks, development of bronchopulmonary dysplasia, and necessity for blood transfusion. No relationship was found with patent ductus arteriosus.

Serum IGF1 measured in the third week post-partum was found to be significantly lower in patients who developed ROP than those who did not and to be independent of gestational age.

Also, IGF1 serum levels at third week of life were significantly lower in those patients who developed bronchopulmonary dysplasia, needed any kind of mechanical ventilation or oxygen support, patent ductus arteriosus, sepsis in the first 3 weeks of life, EPO treatment or needed transfusion. Although IGF1 level was lower in patients with intracranial haemorrhage, it was not significant (Table 2).

**Table 2:** IGF1 levels in the 3^rd^ week of life according to different clinical variables.

	*No*	*Yes*	p
***Mechanical ventilation***	48.23±15.41	37.79±13.95	0.001
***Oxygen administration***	47.38±17.11	36.27±11.85	0.000
***Intracranial haemorrhage***	41.17±14.85	37.57±15.33	NS
***Sepsis (<3 weeks post-partum)***	45.30±15.20	33.68±11.80	0.000
***Patent ductus arteriosus***	42.80±15.29	31.21±9.22	0.000
***Bronchopulmonary dysplasia***	42.59±15.15	29.83±8.21	0.000
***Blood transfusion***	43.26±15.02	29.90±9.05	0.000
***EPO treatment***	46.45±16.75	34.85±10.52	0.000

**Figure 1:**
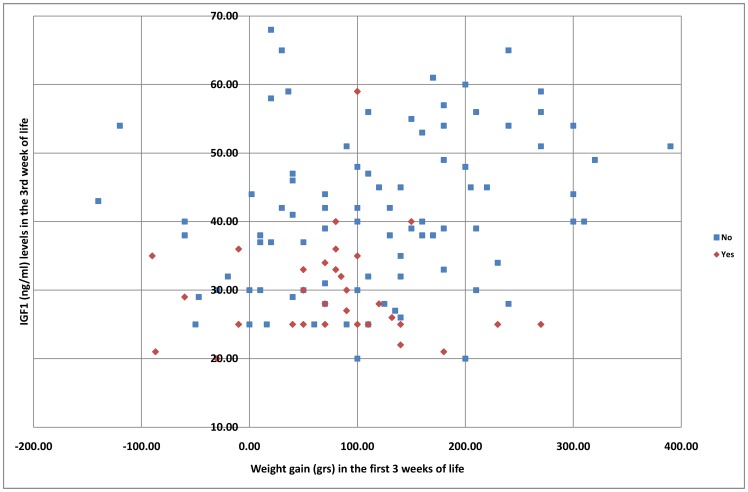
IGF1 levels (Y-axes) dispersion at third week related with the weight gain (X-axes) in the first three weeks of life.

We evaluated the weight gain during the first 3 weeks of life and was correlated with IGF1 levels at third week of life (Figure 1).

**Figure 2:**
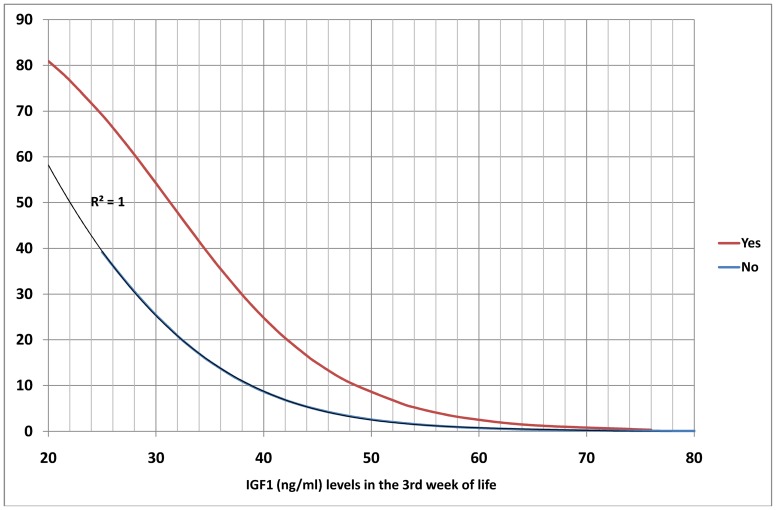
Probability of Retinopathy of Prematurity as a function of IGF1 level and the presence or absence of sepsis in the first three weeks post-partum.

**Figure 3:**
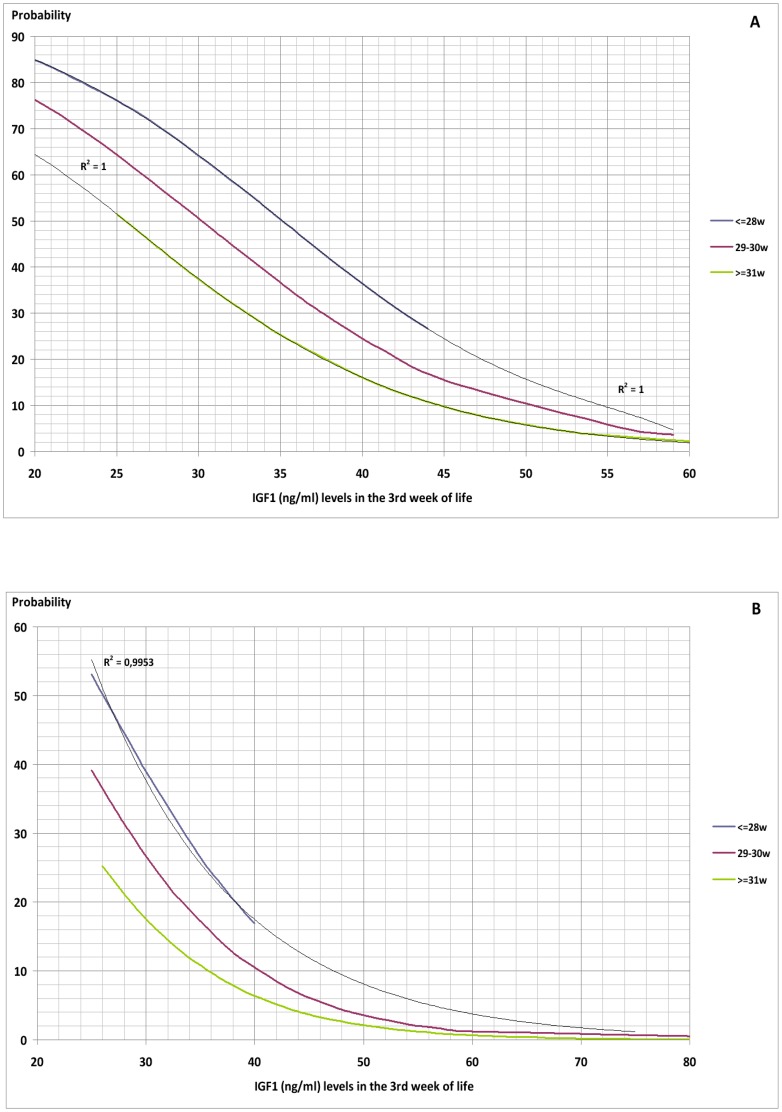
Probability of Retinopathy of Prematurity as a function of IGF1 level, presence of sepsis (A) or not (B) in the first three weeks post-partum and gestational age at birth.

**Figure 4:**
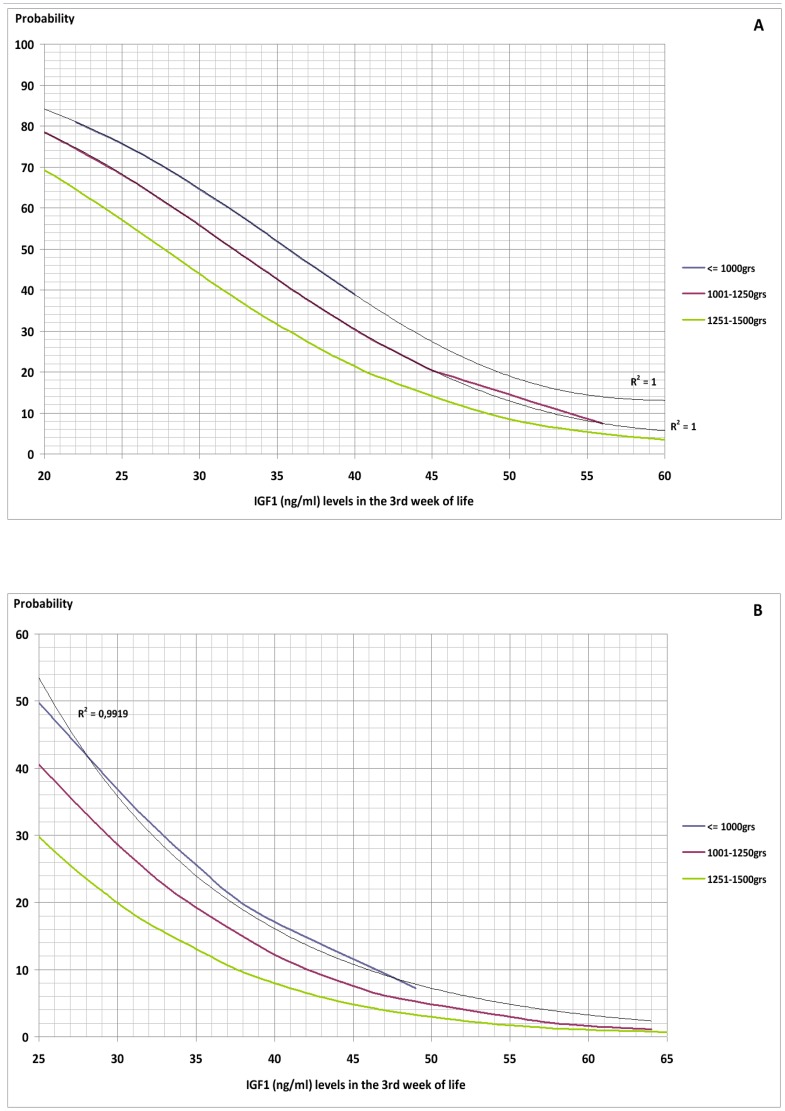
Probability of Retinopathy of Prematurity as a function of IGF1 level, presence of sepsis (A) or not (B) in the first three weeks post-partum and birthweight.

A multiple logistic regression analysis carried out on the variables found by Student's t-test to be significantly correlated with development of ROP showed that only serum levels of IGF1 in the third week (p = 0.002) and presence of sepsis –considered as clinical, analytical and/or microbiological- in the first three weeks (p = 0.036) significantly increased the probability of developing ROP (Table 3) (Figures 2–4).

**Table 3:** Results of multiple logistic regression (p 0.000). Contrast of variables.

		p
***Gestational age at birth***		NS (0.925)
***Birthweight (g)***		NS (0.062)
***Mechanical ventilation***		NS (0.791)
	HFO	NS (0.398)
	Non invasive	NS (0.109)
***Oxygen administration***		NS (0.540)
***Intracranial haemorrhage***		NS (0.079)
***Sepsis (<3 weeks post-partum)***		0.036
***Bronchopulmonary dysplasia***		NS (0.238)
***Blood transfusion***		NS (0.797)
***EPO treatment***		NS (0.371)
***IGF1 (3^rd^ week)***		0.002

The graphs obtained give the probability of developing ROP of any stage with respect to serum IGF1 level in the third week post-partum and presence or absence of sepsis in the first three weeks. Further categorisation by gestational age and birthweight show a greater probability of developing ROP with lower gestational age and weight at birth.

Application of the described screening method to our studied population gave ROC curves for the graphs of gestational age and weight at birth with a discriminative efficiency for ROP diagnosis of 86.9% CI95% (80.4%–93.5%) and 87.3% CI95% (81%–93.6%), respectively. The ROC curves show a cut-off point of 30%. The screening method employed therefore gave a positive result (high risk of developing ROP) if either graph (gestational age or birthweight) showed a probability of ROP of more than 30%, but a negative result (low risk of ROP) if the probability shown by both graphs (gestational age and birthweight) was less than 30%. This method allows discrimination between patients at risk of developing ROP and those not at risk with a sensitivity (S) of 89.2%, specificity (E) of 76.7%, positive predictive value (PPV) of 57.9%, and negative predictive value (NPV) of 95.2%. If, for example, the probability curves are applied to a patient of birthweight over 1250 g and/or more than 30 weeks of gestational age at birth, and a negative result is obtained, it is very likely that the patient will not develop ROP (S 80%, E 96.4%, PPV 66.7%, NPV 98.2%).

## Discussion

We have developed a method for the prediction of retinopathy of prematurity, based on serum levels of IGF1 in the third week and the presence or absence of sepsis in the first three weeks after birth. By sub-dividing the curves of probability of developing the disease obtained, according to gestational age and weight at birth, we have developed a highly discriminative and sensitive means of prediction of ROP in a cohort of 145 newborn premature babies of birthweight <1500 g and/or < 32 weeks gestational age at birth. The usefulness of our preclinical screening method is that it has the ability to reduce the number of patients who would otherwise undergo currently established routine screening, predicting with a high probability whether or not a patient is at risk of developing the disease.

Therefore, the use of a preclinical screening method in premature newborn patients should limit the number of full ophthalmic retinal examinations, as well as making better use of resources – human, technical and economic – while maintaining the current indications for investigation of patients at high risk of developing ROP.

It may be anticipated that universal examination of premature babies of birthweight < 1250 g and/or gestational age at birth of < 30 weeks, together with tailored investigations in individual at-risk patients, identified principally using the screening curves such as those that we have shown here, will optimise available resources while at least maintaining current levels of identification and treatment of ROP [20],[21],[22],[23],[24]. In the same context, the most recent guidelines of the American Academy of Paediatrics recommend the restriction of universal screening to newborn babies of less than 30 weeks gestational age, although maintaining the birthweight cut-off at 1500 g [11].

In the current study, applying our preclinical screening method to patients of more than 1250 g and/or more than 30 weeks gestational age has enabled us to show that a negative test result (curves for gestational age and weight at birth both showing a probability of developing ROP of less than 30%) has an accuracy (NPV) of 98.2%. This high predictive value would mean that a series of ophthalmic investigations could be substituted for a single scan at 40 weeks of corrected gestational age that would serve as validation and ensure the effectiveness of our preclinical screening method.

Other published screening methods are based on post-natal weight gain in premature neonates [14],[15],[16]. Studying IGF1 levels, by contrast, assumes that they give a better indication of the status of the newborn, in terms not only of nutrition, since good nutritional status and potnatal growth are associated with increases in IGF1, but also several other factors such as the development of sepsis, bronchopulmonary dysplasia, the need for erythrocyte transfusion or EPO treatment, and all of which are associated with diminished levels of IGF1. Because of these results, we think that IGF1 is a good marker for the clinical status of the premature baby. Lower IGF1 levels are associated to critical patients who need erythrocyte transfusion, or respiratory support, or develop a bronchopulmonary dysplasia or a patent ductus arteriosus, i.e., situations all related to the genesis of ROP. There are indeed, premature babies with normal post-natal weight gain but low levels of IGF1 who develop ROP. On the other hand, many children have poor weight gain, high IGF1 level and do not develop ROP. Also, other newborns have a great weight gain at the expense of free water instead of lean mass which is not correlated with an increasing level of IGF1. We therefore consider that a screening system based on IGF1 levels results in a better understanding of the true status of the patient.

The other parameter that we have analysed and found to be strongly correlated with the development of ROP is the presence of sepsis in the first three weeks post-partum, as defined by clinical, analytical or microbiological criteria [25]. The importance of sepsis has previously been described [9], while the current study shows that its combination with IGF1 levels increases the accuracy of prediction of ROP.

The introduction to any particular hospital neonatal unit of a screening method for ROP, with its corresponding reduction in the number of criteria used to identify at-risk patients should be accompanied by a validation study in which patients not deemed to be at-risk are shown to have developed normal retinal vascularisation by 40 weeks of corrected gestational age.

## Conclusion

A full understanding of the incidence of ROP in any individual neonatal unit and thus the appropriate introduction of an IGF1 screening method can be used to develop a rigorous and useful system for the identification of high-risk patients. Preclinical screening could reduce the number of patients undergoing ophthalmic examination so lightening the burden on the ophthalmological team and maintaining adequate levels of care quality.
